# Treatment with native heterodimeric IL-15 increases cytotoxic lymphocytes and reduces SHIV RNA in lymph nodes

**DOI:** 10.1371/journal.ppat.1006902

**Published:** 2018-02-23

**Authors:** Dionysios C. Watson, Eirini Moysi, Antonio Valentin, Cristina Bergamaschi, Santhi Devasundaram, Sotirios P. Fortis, Jenifer Bear, Elena Chertova, Julian Bess, Ray Sowder, David J. Venzon, Claire Deleage, Jacob D. Estes, Jeffrey D. Lifson, Constantinos Petrovas, Barbara K. Felber, George N. Pavlakis

**Affiliations:** 1 Human Retrovirus Section, Vaccine Branch, Center for Cancer Research, National Cancer Institute at Frederick, Frederick, Maryland, United States of America; 2 Vaccine Research Center, National Institute of Allergy and Infectious Diseases, Bethesda, Maryland, United States of America; 3 Human Retrovirus Pathogenesis Section; Vaccine Branch, Center for Cancer Research, National Cancer Institute at Frederick, Frederick, Maryland, United States of America; 4 AIDS and Cancer Virus Program, Leidos Biomedical Research, Inc., Frederick National Laboratory for Cancer Research, Frederick, Maryland, United States of America; 5 Biostatistics and Data Management Section, Center for Cancer Research, National Cancer Institute, National Institutes of Health, Rockville, Maryland, United States of America; Emory University, UNITED STATES

## Abstract

B cell follicles in secondary lymphoid tissues represent an immune privileged sanctuary for AIDS viruses, in part because cytotoxic CD8^+^ T cells are mostly excluded from entering the follicles that harbor infected T follicular helper (T_FH_) cells. We studied the effects of native heterodimeric IL-15 (hetIL-15) treatment on uninfected rhesus macaques and on macaques that had spontaneously controlled SHIV infection to low levels of chronic viremia. hetIL-15 increased effector CD8^+^ T lymphocytes with high granzyme B content in blood, mucosal sites and lymph nodes, including virus-specific MHC-peptide tetramer+ CD8^+^ cells in LN. Following hetIL-15 treatment, multiplexed quantitative image analysis (histo-cytometry) of LN revealed increased numbers of granzyme B^+^ T cells in B cell follicles and SHIV RNA was decreased in plasma and in LN. Based on these properties, hetIL-15 shows promise as a potential component in combination immunotherapy regimens to target AIDS virus sanctuaries and reduce long-term viral reservoirs in HIV-1 infected individuals.

**Trial registration:** ClinicalTrials.gov NCT02452268

## Author summary

Heterodimeric interleukin-15 (hetIL-15), the stable native complex of IL-15 and IL-15 Receptor α (IL-15/IL-15Rα), activates and expands cytotoxic T and NK cells. Based on these properties, hetIL-15 is currently being evaluated in clinical trials in advanced cancer. To explore potential utility in AIDS virus infection, we evaluated the effects of hetIL-15 in uninfected rhesus macaque monkeys or monkeys with low levels of chronic viremia after SHIV infection. hetIL-15 treatment increased CD8^+^ lymphocytes within the B cell follicles of lymph nodes and decreased cell-associated viral RNA in LN as well as plasma viremia. These results suggest hetIL-15 may have beneficial effects in chronic HIV infection by allowing CD8+ T cells to access infected cells within otherwise immune privileged sanctuary sites in B cell follicles of secondary lymphoid tissues.

## Introduction

Interleukin-15 (IL-15) is a gamma-chain cytokine essential for the production and maintenance of NK cells and plays an important role in the expansion and long-term preservation of memory T cells [1–7]. IL-15 is produced by stromal cells in several tissues, some blood endothelial cells and antigen presenting cells [8–10]. Although a single-chain form of IL-15 has been produced and evaluated in early stage clinical trials [11], several experiments have suggested that IL-15 *in vivo* acts in concert with a transmembrane polypeptide designated IL-15 Receptor alpha (IL-15Rα) [12–22]. We have characterized the native form of IL-15 as the highly glycosylated heterodimeric cytokine (hetIL-15) formed by the tight association of the single-chain IL-15 with IL-15Rα during production, and we showed that IL-15Rα does not serve a receptor function but rather is an integral component of the natural heterodimeric cytokine [19, 22–24]. The IL-15/IL-15Rα complex is active on the surface of the IL-15 producing cells and is also rapidly shed into plasma upon proteolytic cleavage of the IL-15Rα chain [18, 19, 23, 24]. hetIL-15 has an extended half-life *in vivo* and stimulates proliferation and cytotoxic commitment of NK cells and CD8^+^ effector T cells by binding to the IL-2/IL-15βγ receptor. We have shown that recombinant hetIL-15 induces proliferation, activation and increased cytotoxic potential of lymphocytes and, importantly, induces migration of lymphocytes into tumors in a murine model [25]. Due to these properties and its ability to delay tumor progression in animal models, hetIL-15 has progressed to clinical trials for metastatic cancer (NCT02452268). Studies monitoring the systemic effects of IL-15 in non-human primates using recombinant *E*. *coli*-derived rhIL-15, glycosylated CHO cell produced single-chain IL-15, or ALT-803, a IL-15 mutant Fc fusion protein, showed expansion of memory T cells and NK cells in blood [6, 26–34]. IL-15 has potential applications in the treatment of human diseases where enhanced cytotoxicity is desired, for example in the therapy of cancer and certain chronic infections such as HIV infection.

HIV infection in humans and SIV/SHIV infection in macaques lead to the establishment of long-term viral reservoirs able to persist despite antiretroviral therapy (ART) and give rise to recrudescent infection when treatment is interrupted. Although long-lived latently infected CD4^+^ T cells have been identified as an important contributor to this viral reservoir, it has also been shown, in both HIV infected humans and SIV infected macaques, that cells actively producing virus can persist in privileged anatomic locations including B cell follicles within secondary lymphoid tissues [35–44]. Similar to HIV infected elite controllers, macaques infected with SIV that suppress viremia to low or undetectable levels continue to harbor virus infected cells within the follicles [41, 42]. CD8^+^ T cell depletion increases virus production [45–47], suggesting CD8-dependent immunological mechanisms of control [36, 37, 40–42, 48–52]. Virus is able to persist in infected CD4^+^ T_FH_ at least in part due to the relative inability of cytotoxic CD8^+^ T cells to enter the follicles [52–55]. Most CD8^+^ T cells lack CXCR5, the chemokine receptor that allows CXCL13-guided chemotactic trafficking to the B cell areas in the LN [56]. Interventions that facilitate targeting of infected cells in the follicles will likely be required as part of an effective combinatorial treatment to reduce or eliminate viral reservoirs.

In the present study, we used a two-week hetIL-15 treatment regimen to evaluate the effects in both uninfected macaques and macaques that had spontaneously controlled SHIV infections and maintained low levels of chronic viremia. We investigated the effect of hetIL-15 on lymphocytes of treated macaques using flow cytometry and confocal image analysis of LN. hetIL-15 treatment resulted in increased frequency of granzyme B (GrzB) expressing CD8^+^ T cells in B cell follicles, associated with decreases in cell-associated viral RNA within LN and reduced plasma viral RNA in the SHIV^+^ rhesus macaques. Thus, hetIL-15 treatment shows potential to target LN AIDS virus sanctuaries as part of combinatorial immunotherapy regimens.

## Results

### hetIL-15 increases effector CD8^+^ T cells systemically and in LN

**Fig 1:**
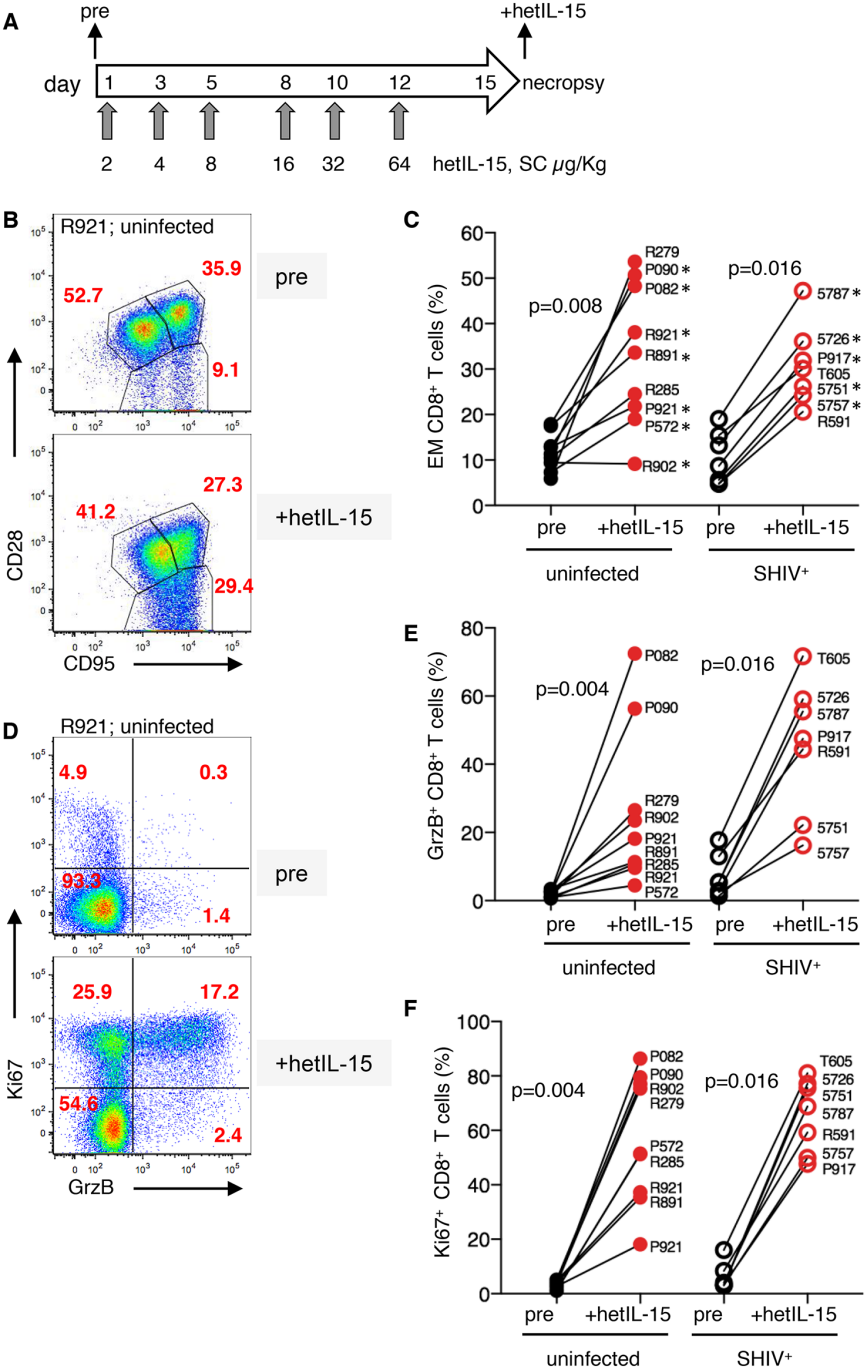
Lymphocyte changes in LN after hetIL-15 treatment. (A) Step-dose regimen of six SC hetIL-15 administrations in rhesus macaques. LN, blood and mucosal tissue lymphocytes were analyzed before (pre) and after treatment (+hetIL-15). Flow cytometry dot plots of LN mononuclear cells show (B) the frequency of CD8^+^ memory subsets, naïve (T_N_, CD28^+^CD95^low^), central memory (T_CM_, CD28^high^CD95^+^) and effector memory (T_EM_, CD28^-^CD95^+^), and (D) granzyme B content and cycling status (GrzB^+^Ki67^+^) from a representative uninfected macaque (R921) upon hetIL-15 treatment. Graphs (C, E, F) summarize results of 16 macaques treated with hetIL-15 of (C) frequency of effector memory CD8^+^ T cells, (E) CD8^+^GrzB^+^ T cells, and (F) cycling (Ki67+) CD8^+^ T cells. Analysis was performed on LN of 9 uninfected animals (filled symbols) and 7 SHIV^+^ macaques (open symbols). Black symbols, pre; red symbols, +hetIL-15. P values are from paired Wilcoxon signed rank test. The 12 animals that were also analyzed for hetIL-15 effects in blood and mucosal tissues (Figs 2 and 3) are indicated by *.

To study the effects of hetIL-15, we applied a two-week treatment protocol to 9 uninfected rhesus macaques (Table 1). The treatment consisted of 6 subcutaneous doses over two weeks with increasing quantities of recombinant hetIL-15 (2–64 μg/kg, Fig 1A, “step-dosing”). This regimen was designed based on the homeostatic nature of hetIL-15 [57, 58], and greatly expands lymphocyte numbers in rhesus macaques while minimizing toxicity. Treatment with this hetIL-15 regimen was well tolerated without clinically apparent toxicity or significant abnormalities in blood chemistry measurements. Both human and macaque hetIL-15 were used in this study as specified in Table 1. These purified cytokines were shown to be equipotent in their ability to induce proliferation of macaque primary CD4^+^ and CD8^+^ T cells and NK cells *in vitro* (S1 Fig).

**Table 1:** Macaques treated SC with hetIL-15.

Animal ID	Sex	Infection status^a^	Infection route	Length of infection (months) ^b^	PVL^c^	hetIL-15 source^d^
P572	F	uninfected	N/A	N/A	N/A	human
R902	F	uninfected	N/A	N/A	N/A	human
R891	F	uninfected	N/A	N/A	N/A	macaque
R921	F	uninfected	N/A	N/A	N/A	macaque
P082^e^	M	uninfected	N/A	N/A	N/A	human
P090^e^	M	uninfected	N/A	N/A	N/A	human
P921	F	uninfected	N/A	N/A	N/A	human
R279	M	uninfected	N/A	N/A	N/A	macaque
R285	M	uninfected	N/A	N/A	N/A	macaque
T605	F	SHIV-CH505 (clade C)	vaginal	6	2200	macaque
T416	F	SHIV-1157ipd3N4 (clade C)	rectal	14	40	macaque
T422	M	SHIV-1157ipd3N4 (clade C)	rectal	13	3600	macaque
T413	F	SHIV-1157ipd3N4 (clade C)	rectal	7	950	human
T419	M	SHIV-1157ipd3N4 (clade C)	rectal	7	150	human
T421	M	SHIV-1157ipd3N4 (clade C)	rectal	7	400	human
T427	M	SHIV-1157ipd3N4 (clade C)	rectal	6	25	human
5751	F	SHIV-327C (clade C)	rectal	10	880	human
5757	F	SHIV-327C (clade C)	rectal	10	480	human
5726	F	SHIV-327C (clade C)	rectal	10	30	human
5787	F	SHIV-327C (clade C)	rectal	10	50	human
R591	M	SHIV-SF162P3 (clade B)	rectal	5	200	macaque
P917	F	SHIV-SF163P3 (clade B)	rectal	45	380	human
P934^f^	F	SHIV-SF162P3 (clade B)	rectal	29	3	human
P995^g^	F	SHIV-SF162P4 (clade B)	rectal	14	<2	human

^a^ Indicated animals were chronically infected with one of four SHIVs with clade B or C Env

^b^ median time of SHIV infection 10 months at day 0 (range 5–45 months)

^c^ plasma virus load (PVL) in RNA copies/ml at day 0 (pre)

^d^ human and macaque hetIL-15 have equipotent activity *in vitro* in macaque cells (S1 Fig). Eight of 24 animals received macaque hetIL-15

^e^ macaques with MamuA*01^+^ MHC class I haplotype

^f^ received high dose-escalation treatment (5–120 μg hetIL-15/kg)

^g^ received a two-week fixed dose treatment 50 μg hetIL-15/kg

**Fig 2:**
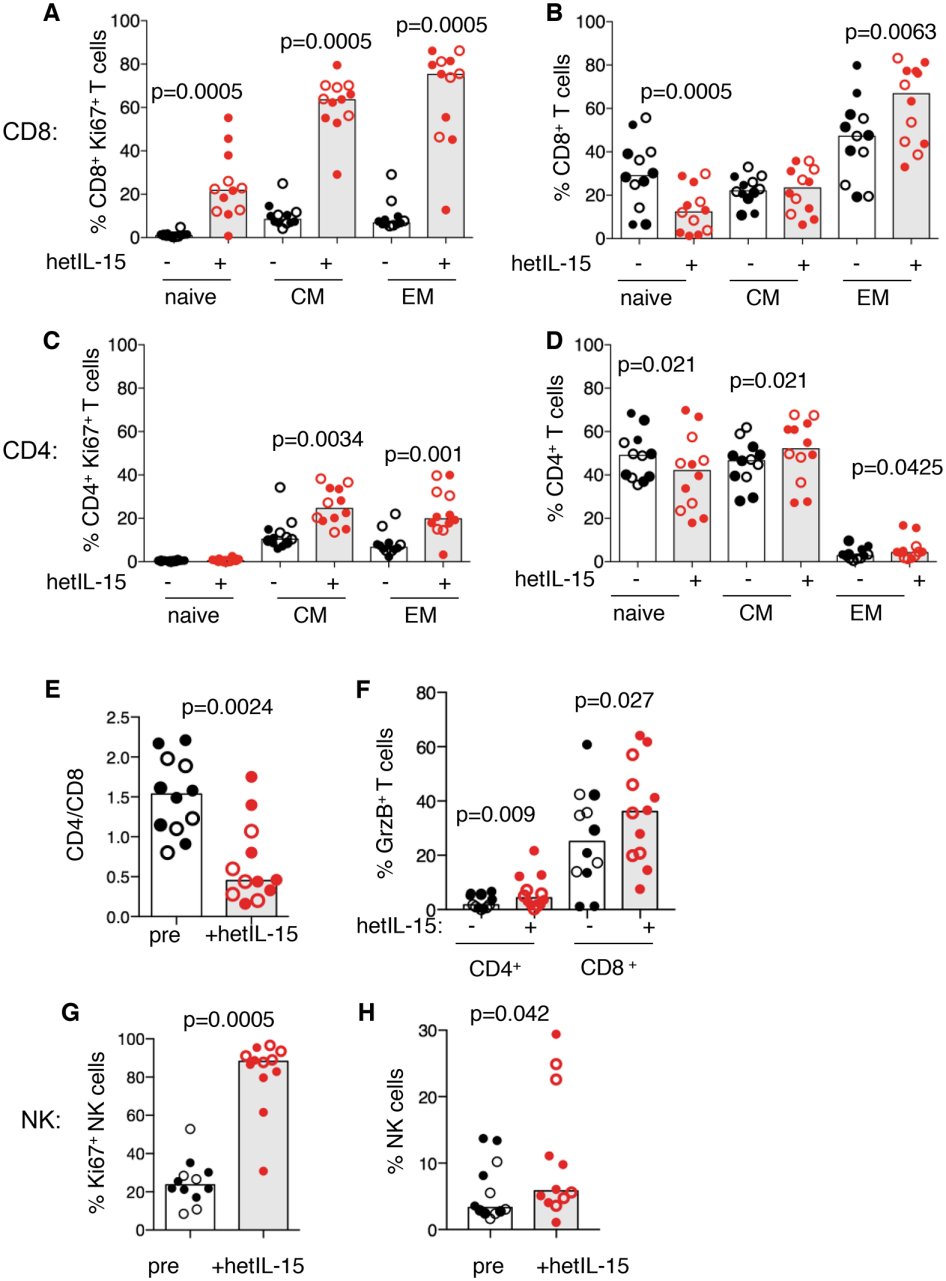
hetIL-15 effects in lymphocytes in peripheral blood. (A) Changes in lymphocyte populations were analyzed in blood samples collected from 12 macaques before (black symbols) and after hetIL-15 administration (red symbols). The animals included are indicated by * in Fig 1C and represent 12 of the 16 animals shown in Fig 1. The effects of hetIL-15 treatment on (A) CD8^+^ Ki67^+^ T lymphocytes; (B) frequency of CD8^+^ subsets; (C) CD4^+^ Ki67^+^ T lymphocytes; (D) frequency of CD4^+^ subsets. (E) Effect of hetIL-15 on the blood CD4/CD8 ratio. (F) Effects of hetIL-15 on the granzyme B content of CD4 and CD8 cells in blood. (G-H) NK (CD3^-^CD16^+^GrzB^-/+^) cells were analyzed by measuring cycling status (Ki67 expression; G) and frequency (H). p values are from paired Wilcoxon signed rank test.

**Fig 3:**
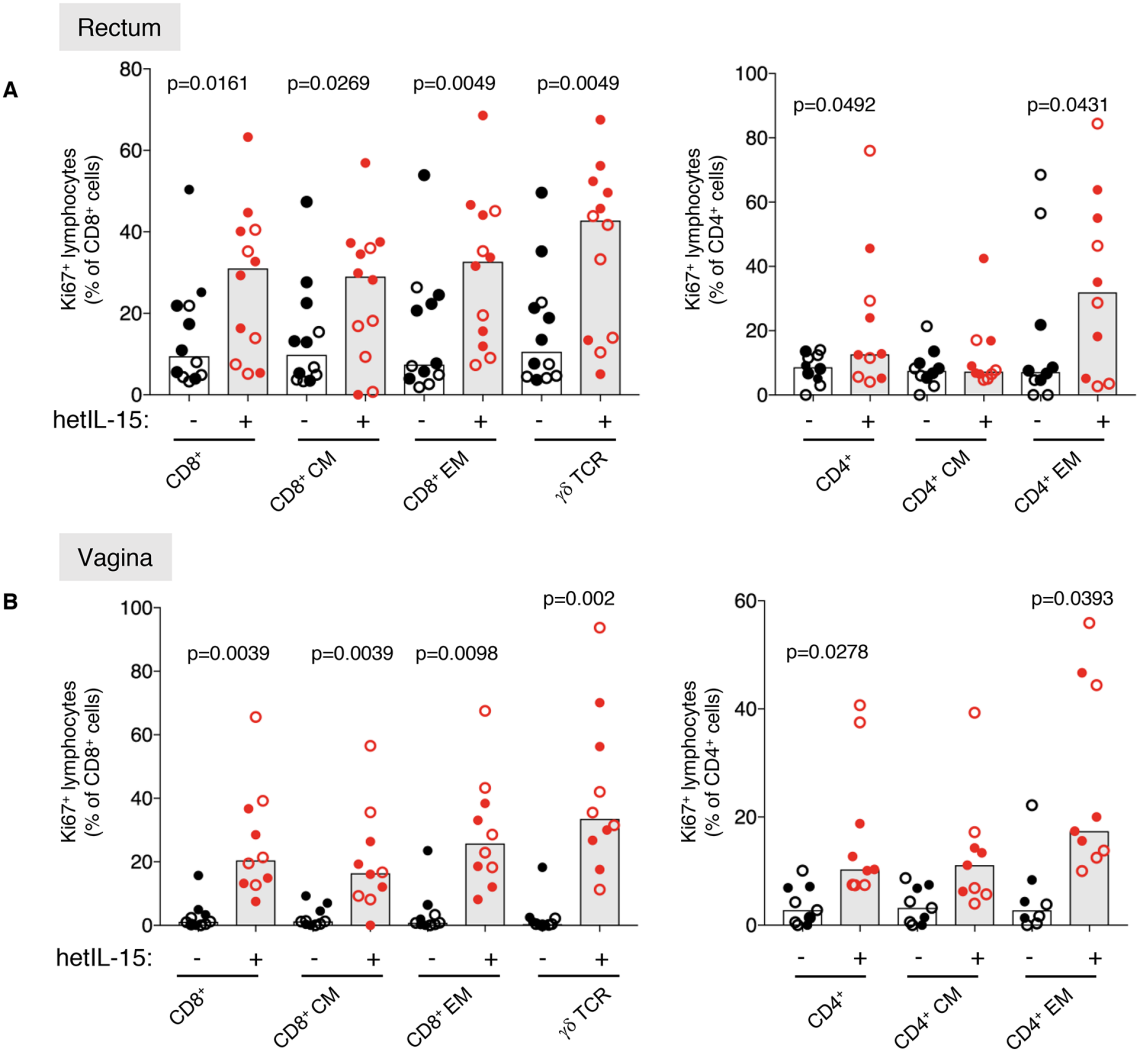
hetIL-15 effects in mucosal effector sites. Analysis of the hetIL-15 effects on lymphocytes from mucosal sites, collected from the same animals shown in Figs 1 and 2. Rectal (N = 12) and vaginal (N = 10) biopsies were obtained before and after hetIL-15 treatment. The mucosal samples were analyzed for changes in Ki67 expression on T cell subsets. The plots show Ki67 levels on T_CM_ (CD95^+^CD28^high^), T_EM_ (CD95^+^CD28^low^) and CD8^+^ T cells expressing the γδ TCR (left panels) and CD4+ T_CM_ and T_EM_ (right panels) in rectal (N = 12) (A) and vaginal (B) (from the 10 female macaques) samples collected before (black symbols) and after hetIL-15 treatment (red symbols). p values are from paired Wilcoxon signed rank test.

Lymph nodes (LN) (Fig 1), blood (Fig 2), and mucosal samples (Fig 3), collected before the first injection (pre) and 3 days after the last hetIL-15 injection, were analyzed by flow cytometry using the gating strategy shown in S2 Fig. As shown in the flow cytometry plots from a representative macaque (R921) in Fig 1B, with group data summarized in Fig 1C, hetIL-15 significantly increased the relative frequency of effector CD8^+^ T cells (T_EM_, CD28^-^CD95^+^) in LN mononuclear cells (LNMC) in all 9 uninfected rhesus macaques (filled symbols). The frequencies of cycling (Ki67^+^) CD8^+^ T cells and cells expressing GrzB, measured in the same 9 macaques, were also significantly increased in LNMC (Fig 1D, 1E and 1F).

To study the effects of hetIL-15 in the setting of chronic virus infection, we analyzed hetIL-15 treatment effects on 7 chronically SHIV-infected rhesus macaques that had spontaneously controlled their infections (Table 1). The SHIV^+^ macaques were selected based on their low persistent plasma viral loads and were asymptomatic throughout the chronic period of infection. At treatment initiation, the animals had been infected for a median of 9 months (range 5–45 months) with either clade B or C SHIV (Table 1). The selection of these otherwise asymptomatic SHIV-infected macaques allowed examination of effects on both immunological and virological parameters upon hetIL-15 treatment.

We applied the two-week hetIL-15 treatment protocol to these SHIV^+^ macaques, collected the same samples (LN, blood, mucosal tissues) and performed the analysis described above for the uninfected macaques (Figs 1, 2 and 3; open symbols). Treatment with this regimen was equally well tolerated in these SHIV^+^ macaques as in uninfected animals. Analysis of LN samples showed that hetIL-15 treatment had a significant effect on the frequency of LNMC T_EM_ CD8^+^ cells (Fig 1C), GrzB^+^ CD8^+^ T cells (Fig 1E) and the cycling status of CD8^+^ T cells (Fig 1F) of the SHIV^+^ macaques (open symbols). Thus, hetIL-15 treatment had significant and similar effects on LNMC in both uninfected and SHIV^+^ animals. In contrast to the T_EM_ CD8^+^ LNMC, no significant change in the distribution of CD4^+^ T cells (naïve, CM, EM) was found within the LN after hetIL-15 treatment (S3 Fig).

To complement the analysis of LNMC, the effects of hetIL-15 on blood lymphocytes were examined (Fig 2) in 12 of the 16 treated animals (indicated by * in Fig 1C). hetIL-15 treatment had significant effects on the cycling status and frequencies of different memory differentiation subsets of CD8^+^ (Fig 2A and 2B) and CD4^+^ (Fig 2C and 2D) T lymphocytes and NK cells (Fig 2G and 2H) in blood of both uninfected (N = 7; filled symbols) and SHIV^+^ (N = 5; open symbols) macaques. These data are in general agreement with previous reports on blood lymphocytes using IV or SC administered single-chain IL-15 or the mutant IL-15 fusion protein, ALT-803 [6, 27, 30, 32–34, 59–61]. hetIL-15 treatment resulted in the highest levels of Ki67^+^ in effector CD8^+^ T cells (CD28^-^CD95^+^), followed by central memory and naïve CD8^+^ T cells (Fig 2A). hetIL-15 also increased the percentage of total T_EM_ cells and decreased the percentage of naïve (T_N_, CD28^+^CD95) cells within the circulating CD8^+^ T cell pool (Fig 2B).

We also analyzed the effect of hetIL-15 on the circulating CD4^+^ T cells (Fig 2C and 2D). hetIL-15 treatment increased the frequency of cycling (Ki67^+^) CD4^+^ T_CM_ (CD28^+^CD95^+^) and T_EM_ cell subsets, but had minimal effect on frequency of different CD4^+^ T cell memory subsets (Fig 2D). The net effect of hetIL-15 in blood was a significant decrease in the CD4/CD8 ratio (Fig 2E). hetIL-15 stimulated cell cycling and GrzB production in both CD8^+^ and CD4^+^ T cells, consistent with induction of a cytotoxic phenotype (Fig 2F). A significant increase in the frequency of NK cells, which, like the EM CD8^+^ T cells, also showed greatly increased expression of Ki67, was also found (Fig 2G and 2H).

HetIL-15 effects on cell populations at mucosal sites were examined in the same macaques after rectal (12 macaques) and vaginal biopsies (10 females) (Fig 3). The effects of hetIL-15 treatment were similar in uninfected (filled symbols) and SHIV^+^ (open symbols) macaques. hetIL-15 treatment significantly increased the frequency of cycling lymphocytes with increased frequencies of Ki67^+^ cells seen in both CD95^+^CD28^high^ T_CM_ and CD95^+^CD28^low^ T_EM_ subsets of CD8^+^ T cells, and in lymphocytes expressing γδ TCR in rectal (Fig 3A, left panel) and vaginal tissues (Fig 3B, left panel). In both mucosal sites, the highest frequencies of Ki67^+^ cells were found among CD95^+^CD28^low^ T_EM_ cells and, especially, γδ T cells (Fig 3A and 3B). Similar to the CD8^+^ T cells, an increase in cycling CD4^+^ T lymphocytes was observed in both mucosal sites (Fig 3A and 3B, right panels) and this increase was mainly driven by the increase of Ki67 among the CD95^+^CD28^low^ T_EM_ CD4^+^ T cells. In aggregate, these results show that subcutaneous administration of hetIL-15 at this dosing schedule affected lymphocyte cycling and cytotoxic potential in different compartments, including LN, blood, and mucosal effector sites.

### hetIL-15 increases cytotoxic cells in B cell follicles

**Fig 4:**
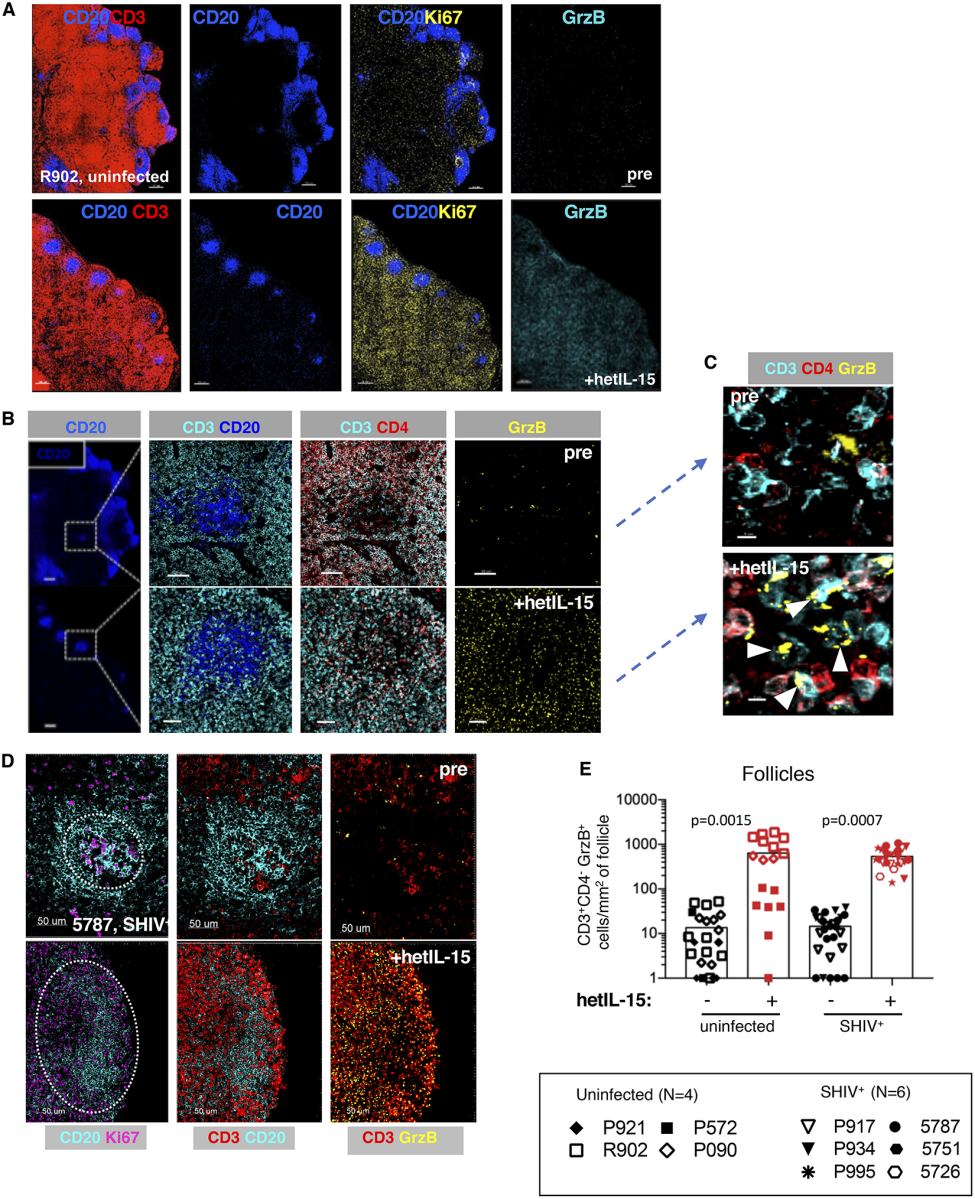
Accumulation of Ki67^+^ and GrzB^+^CD8^+^ T cells in follicular areas upon hetIL-15 treatment. (A) Representative confocal images showing the distribution of CD20 (blue), CD3 (red), Ki67 (yellow) and GrzB (cyan) positive cells in peripheral LN from an uninfected macaque (R902) before and after hetIL-15 treatment. Pre-treatment is shown in the upper panels and +hetIL-15 in lower panels throughout. Follicles are defined as CD20^hi-dim^ areas. (B) Higher magnifications from the same LN in panel A of areas indicated by squares. CD20 (blue), CD3 (cyan), CD4 (red) and GrzB (yellow). Follicular areas defined by CD20 (left panel) show increased presence of CD8^+^ cells (defined as CD3^+^CD4^-^) (middle panel) and GrzB^+^ cells (right panel) upon hetIL-15 treatment. (C) Higher magnification (from panel B) shows presence of CD3^+^ CD4^-^ GrzB^+^ (GrzB^+^CD8^+^) cells upon hetIL-15 treatment (white arrowheads). (D) Confocal images showing the distribution of CD20 (blue), Ki67 (purple), CD3 (red) and GrzB (yellow) positive cells in a representative follicle. Pre (upper panels) and +hetIL-15 (lower panels) from an SHIV^+^ macaque (5787). (E) Individual follicles were analyzed upon hetIL-15 treatment in LN from 4 uninfected (3 animals pre and +hetIl-15; one animal +hetIL-15) and 6 SHIV^+^ macaques (3 animals pre and +hetIl-15; 3 animals +hetIl-15). A range of 2 to 14 follicles were analyzed per animal. The numbers of CD3^+^CD4^-^GrzB^+^ cells per mm^2^ of area are in individual follicles for each animal are shown. Values of 0 were entered as 1 for the graph display only. Bars indicate average values. p values are calculated by two-way ANOVA for the SHIV^+^ versus uninfected and pre- versus post-hetIL-15 effects, with random effects to account for the clustered values by animal.

Since LN are sites of persistent HIV/SIV infection, at least in part due to the inability of most CD8^+^ cytotoxic T cells to effectively access infected T_FH_ in B cell follicles, we evaluated the localization of CD8^+^ T lymphocytes within LN before and after hetIL-15 treatment using multispectral confocal imaging in combination with histo-cytometry (S4 Fig). This technology provides quantitative and spatial information on the phenotype and location of cells in tissues [55, 63]. LN sections were stained with fluorophore-labeled Abs and a nuclear dye (JOJO-1, S4 Fig), and visualized by confocal fluorescent microscopy (Fig 4). Use of formalin-fixed, paraffin-embedded tissues (FFPE) preserves tissue architecture but precludes the use of available antibody clones reactive with rhesus macaque CD8. For this reason, we measured CD3^+^CD4^-^ cells in LN as a surrogate for CD8^+^ T cells. Flow cytometry analysis showed that more than 90% of CD3^+^CD4^-^ lymphocytes in LNMC express CD8 (91.1% +/-3.6 StdDev, average of 20 determinations before and after hetIL-15 treatment), supporting the use of CD3^+^CD4^-^ measurement as a surrogate for CD3^+^CD8^+^ cells. Consistent with the flow cytometry analysis of LNMC, imaging analysis of FFPE LN samples demonstrated increased numbers of Ki67^+^ and GrzB^+^ cells throughout the LN after hetIL-15 treatment (compare top and bottom panels in Fig 4A–4D) as shown in a representative uninfected macaque (R902) and a SHIV^+^ (5787) macaque. This increase was found throughout the LN sections, including the follicular areas (Fig 4B and 4D). Higher magnification views show co-localization of GrzB primarily with CD3^+^CD4^-^ (CD8) T cells (Fig 4C, white arrowheads).

Quantitative analysis using histo-cytometry was performed on individual follicles from 4 uninfected and 6 SHIV^+^ macaques (Fig 4E). A histo-cytometry software platform was used to project the follicular areas in a 2D plot format using the CD20 intensity (CD20^hi/dim^, see S4 Fig). The same criteria were applied to all analyzed tissues. Follicular areas localized in areas displaying tissue fragmentation or insufficiently resolved due to planar limitations that prevented an accurate follicular mapping were not used for our analysis. hetIL-15 treatment resulted in significantly increased numbers of GrzB^+^ CD8^+^ T cells/mm^2^ in the follicles (Fig 4E). The low numbers of GrzB^+^ CD8^+^ T cells/mm^2^ before treatment are consistent with previous findings in HIV infected humans and SIV infected macaques that cytotoxic CD8^+^ T cells accumulate within lymphoid tissues, but not within follicles [52–55]. These results demonstrate that hetIL-15 treatment can facilitate localization of GrzB^+^CD8^+^ T cells in follicles in both uninfected and SHIV^+^ macaques. Therefore, hetIL-15 treatment increases the number of GrzB^+^ lymphocytes not only in peripheral effector sites, but also in LN follicles, a well-documented sanctuary site for HIV/SIV [35–40, 64]. This increase in GrzB^+^CD8^+^ cell numbers in B cell follicles raised the possibility that productively infected cells within follicles may become more accessible to immune surveillance.

Since hetIL-15 drives cytotoxic cells into areas of persistent HIV/SIV reservoirs, we examined its effect in two SIV-infected macaques rendered aviremic by cART treatment. hetIL-15 step-dose treatment was well tolerated and resulted in CD8^+^GrzB^+^ cell infiltration of the LN (S5 Fig). The animals remained aviremic during and after treatment. These results show that macaques infected with SIV and treated with cART can be treated with hetIL-15 and accumulate large numbers of cytotoxic CD8^+^ lymphocytes into the areas of persistent reservoirs.

### hetIL-15 increases SIV-specific CD8^+^ T cells within peripheral LN

**Fig 5:**
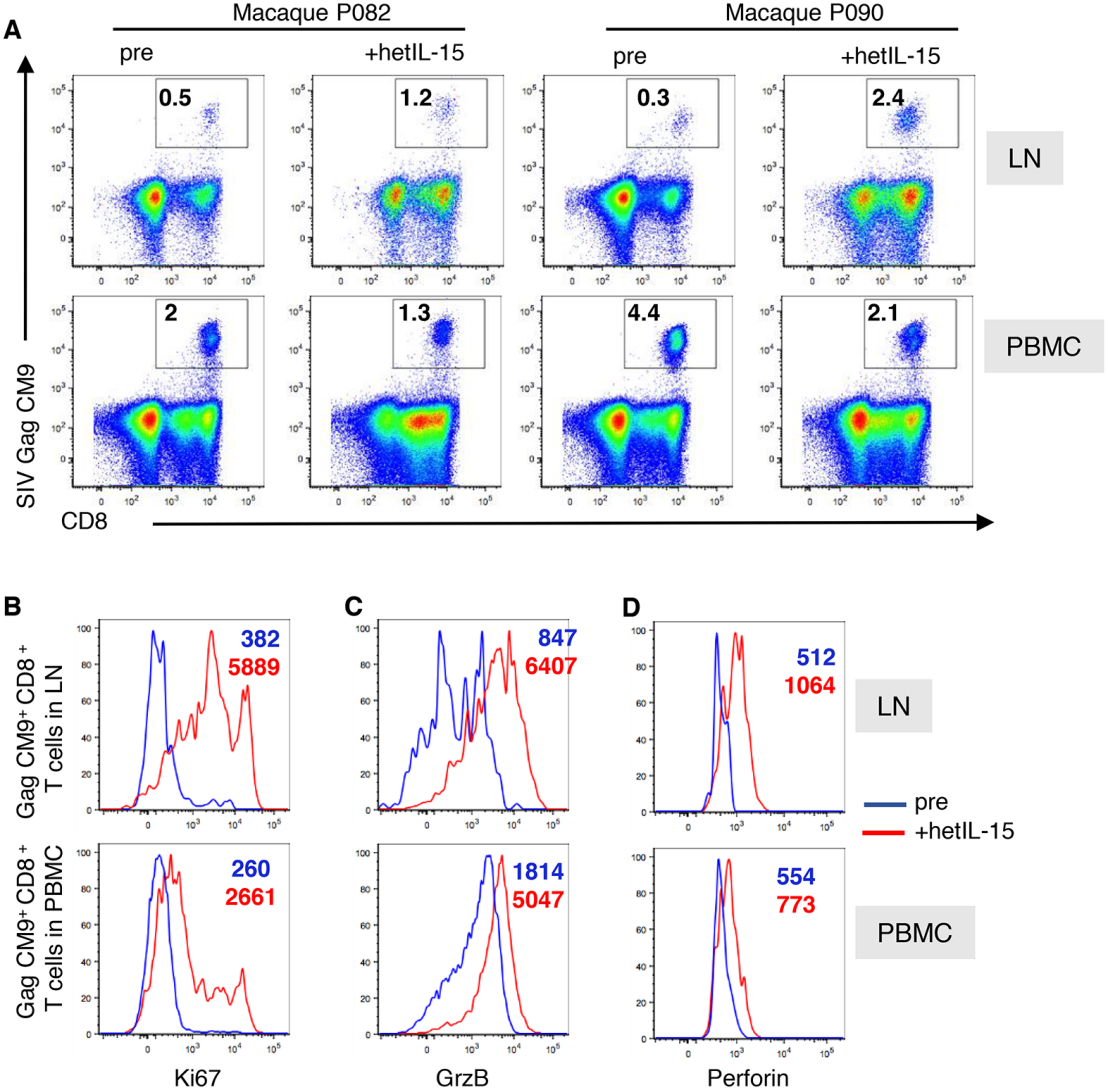
Effects of hetIL-15 on SIV-specific CD8^+^ T cells in peripheral blood and LN of two *gag* DNA vaccinated MamuA*01^+^ macaques. (A) Dot plots showing changes in the frequency of Gag CM9-specific CD8^+^ T cells in axillary LN and PBMC upon hetIL-15 treatment of macaques P082 and P090. These animals received a SIV *gag* DNA vaccine. The CM9-tetramer responses are expressed as % of total T cells. For staining, the PE-conjugated CM9 tetramer (MBL International Corporation) was added to the samples 5 min prior to the addition of the antibody cocktail for surface staining. (B, C, D) Histogram overlays show increased levels of Ki67 (B), GrzB (C) and perforin (D) in Gag CM9-specific T cells upon hetIL-15 treatment in LN and PBMC. pre, blue line, +hetIL-15, red line. Numbers within the histogram overlays show the mean fluorescent intensity (MFI) for the specific markers.

We next used flow cytometry to examine whether hetIL-15 treatment could increase, in addition to the total CD8^+^ T cell population, the frequency of virus-specific lymphocytes within the LN. We analyzed two uninfected MamuA*01^+^ animals (P082 and P090, Table 1) that had received prior an SIV *gag* DNA vaccine [62]. Gag CM9-specific CD8^+^ T cells were detected in LNMC and blood by MHC-peptide/tetramer analysis of both animals prior to treatment (Fig 5). hetIL-15 treatment increased (2.5 to 8-fold) the frequency of CM9^+^ CD8^+^ T cells in LNMC (0.5% to 1.2% in P082; 0.3% to 2.4% in P090) (Fig 5A, upper panels). This increase was associated with a decrease of Gag CM9^+^ CD8^+^ T cell frequency in the circulation (Fig 5B, lower panels), suggesting that the change in SIV-specific CD8^+^ T cells within the LN may have reflected changes in lymphocyte trafficking. The Gag CM9-specific CD8^+^ T cells detected in both LNMC and blood showed increased Ki67^+^ (Fig 5B), granzyme B (Fig 5C) and perforin expression (Fig 5D), compared to matched pre-treatment samples, indicative of an activated, proliferative phenotype with cytotoxic potential. Thus, hetIL-15 treatment not only increased total CD8^+^ effector T cells (Fig 1) but also SIV-specific effector T cells in LN.

### hetIL-15 enhances functional responses in SIV-specific CD8^+^ T cells

**Fig 6:**
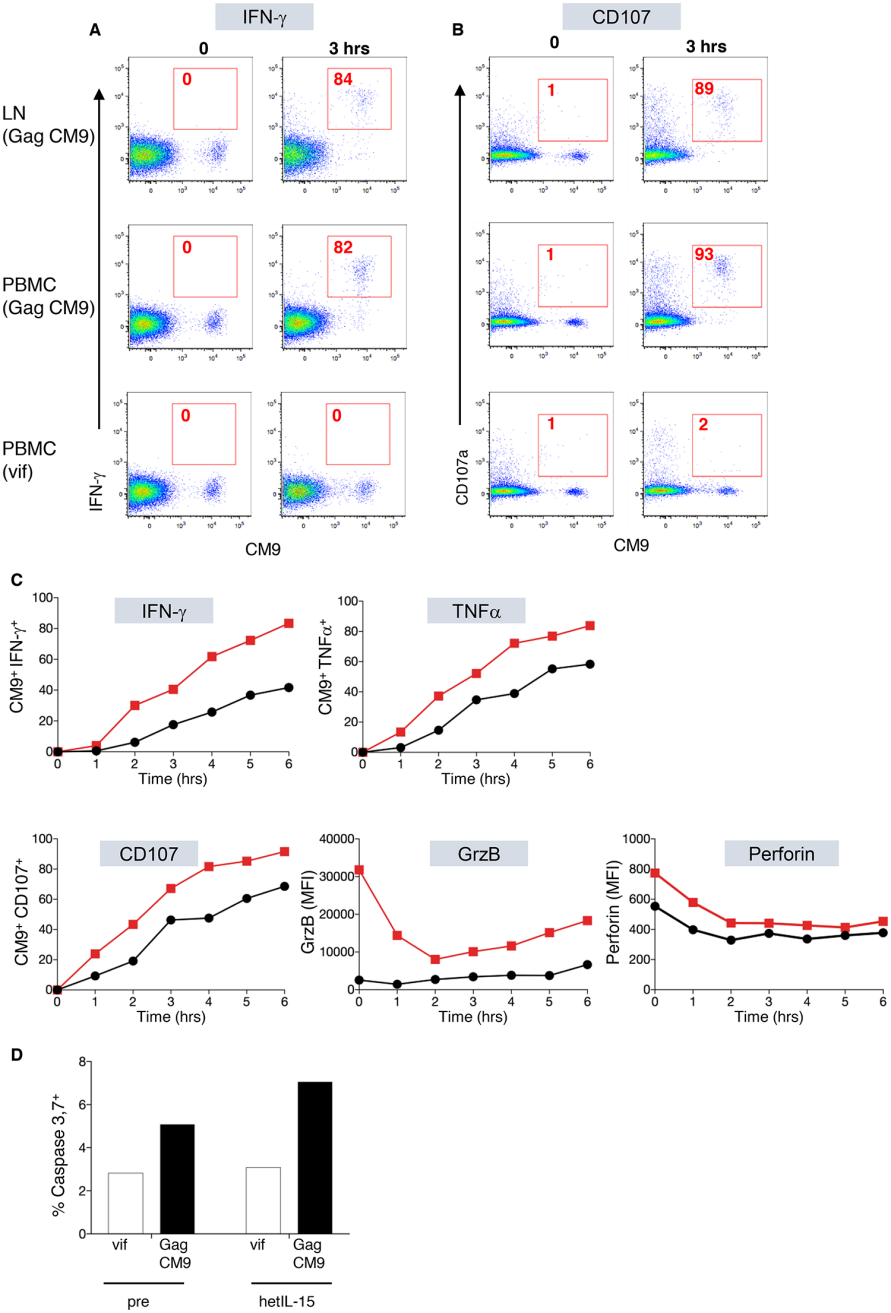
Functional assays for CM9-induced SIV-specific responses. Dot plots show production of IFN-γ (A) and degranulation CD107 (B) by CD8^+^ T cells specific for the MamuA01 restricted Gag CM9 epitope upon specific stimulation of the TCR with the CM9 peptide. The plots show the functional responses at 0 and 3 hours by lymphocytes from LNMC and PBMC of macaque P082. Similar data were obtained from macaque P090. (C) Time course showing the frequency of IFN-γ, TNFα and CD107 positive CD8^+^ CM9^+^ T cells in blood, as well as changes in their content of granzyme B and perforin after stimulation with the CM9 peptide in lymphocytes recovered before (black symbols) and after (red symbols) *in vivo* hetIL-15 treatment. Results in (C) are from a separate experiment with similar results to that shown in panels A and B. (D) Graph showing the frequency of bone marrow cells (macaque P090) loaded with the CM9 peptide or a peptide pool covering HIV-1 Vif undergoing apoptosis (measured as caspase 3 and 7^+^ cells) after incubation with LNMC obtained before and after hetIL-15 treatment.

We also analyzed whether the expanded SIV-specific CD8^+^ T cells had preserved functional properties after hetIL-15 treatment. We monitored at different time points the production of IFN-γ and TNFα, as well as the release of cytotoxic granules measured by CD107 (Fig 6A and 6B) on T lymphocytes stimulated with the MamuA*01-restricted SIV Gag_181-189_ peptide from both PBMC and LNMC isolated before and after hetIL-15 treatment of the two MamuA*01^+^ macaques shown in Fig 5. The data from macaque P082 are shown in Fig 6. The cytokines were rapidly produced by the CM9-specific CD8^+^ T cells in both PBMC and LNMC. Simultaneously to the cytokine production, the cells became CD107^+^. In contrast to these results, no functional responses, neither cytokine production nor degranulation were observed in any of the samples after stimulation with an unrelated Vif peptide pool (Fig 6A and 6B, bottom plots). These data demonstrate that the expanded CM9^+^ CD8^+^ T cells were able to respond by cytokine production and release of cytotoxic granules upon specific TCR stimulation by their cognate antigen, which is the hallmark of cytotoxic T cells. In a different experiment, we also compared the kinetics of these responses in samples collected before and after *in vivo* hetIL-15 treatment (Fig 6C). We found that, as early as 1 hour upon specific TCR stimulation, the samples obtained after hetIL-15 treatment had higher levels of IFN-γ and TNFα production and were already actively degranulating (CD107^+^) as demonstrated also by the decrease in the intracellular content of both granzyme B and perforin, and these differences were maintained during the six hours of monitoring after stimulation. Like in previous experiments (Fig 6A and 6B, bottom plots), none of these responses were found in samples treated with the unrelated Vif peptide pool.

Finally, to monitor the cytotoxic potential against virus-expressing cells, we performed a killing assay using autologous CFSE-labeled bone marrow cells loaded with the CM9 peptide as targets. Target cells and unsorted LNMC were mixed at a ratio 1:1 and active killing among the CFSE population was monitored by flow cytometry after adding the fluorescent reagent 660-DEVD-FMK to the samples. This compound actively enters the cells and binds irreversibly to the active site of caspase 3 and 7, the two main caspases activated by granzyme B. We found similar levels of caspase 3 and 7 in cells loaded with the unrelated Vif peptide pool and exposed to LNMC taken either before or after hetIL-15 treatment (Fig 6D). In contrast, the frequency of cells actively undergoing apoptosis was clearly increased in the samples loaded with the specific CM9 peptide and this increase was higher when LNMC recovered after hetIL-15 treatment were used as effectors (Fig 6D). Taken together these data demonstrate that hetIL-15 enhances the cytolytic responses by CD8^+^ T cells upon specific TCR stimulation.

### hetIL-15 treatment reduced viral RNA in LN and plasma of SHIV^+^ macaques

**Fig 7:**
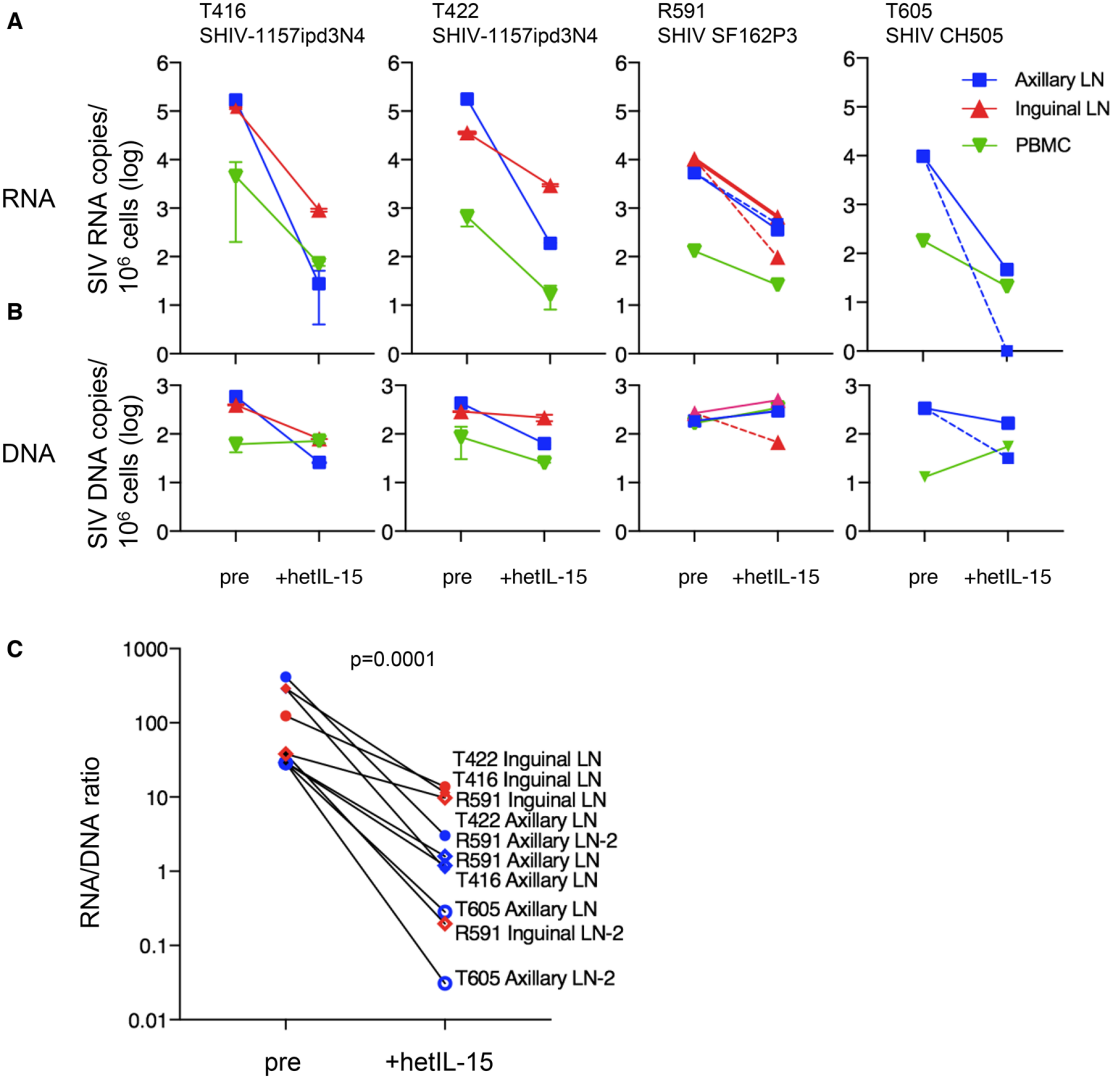
hetIL-15 treatment reduces the viral burden. (A) Quantitation of cell-associated viral RNA in axillary and inguinal LN and PBMC of 4 SHIV^+^ macaques (T416, T422, R591, T605) upon hetIL-15 treatment. The viral RNA determinations in T416 and T422 are from duplicate measurements from purified flash-frozen LNMC and PBMC. The viral RNA determinations in R591 and T605 are from single measurements of flash-frozen LN before treatment and from two independent flash-frozen LN collected after hetIL-15. Dotted and solid lines denote the two independent LN samples. No inguinal LN was available for T605 post treatment. Axillary LN (blue square), inguinal LN (red triangle) and PBMC (green triangle) data are shown. (B) Quantitation of viral DNA copies from the samples of the macaques in panel A. (C) RNA/DNA ratio of the measurements shown in panels A and B. T416, filled diamond; R591, open diamond; T422, filled circle; T605, open circle. RNA/DNA ratio are for inguinal LN (red symbols) and axillary LN (blue symbols). p value for the difference before and after treatment determined by Mann-Whitney test.

To further characterize the effects of hetIL-15, cell associated viral RNA was measured in PBMC and LN by quantitative RT-PCR before and after hetIL-15 treatment in 4 macaques (Fig 7). These animals were infected with and spontaneously controlled clade B or C SHIVs as indicated. These measurements showed significant decreases of cell-associated viral RNA in axillary and inguinal LN (p = 0.0001, Mann-Whitney test) (Fig 7A). The decrease was more prominent in axillary (range 11−10^3^ fold) than inguinal LN (range 12−10^2^ fold) or PBMC (3−10^2^ fold). Viral DNA levels were not appreciably affected by hetIL-15 treatment (Fig 7B). Thus, viral RNA/DNA ratios decreased significantly upon treatment (Fig 7C) in all samples (p = 0.0001, Mann-Whitney test). The differential effects of hetIL-15 treatment on viral RNA and viral DNA are consistent with the observation that a substantial fraction of DNA PCR signal for SIV gag likely reflect proviral genomes that are not replication competent, due to deletions and/or hypermutations [65], while virus specific CTL are expected to only affect cells capable of expressing viral antigens.

We also measured plasma VL in 13 SHIV^+^ macaques treated with the same two-week hetIL-15 protocol, including the animals in Fig 7A above (Table 2). The median time of SHIV infection was 10 months (range 5–45 months) at the time of initiation of hetIL-15 treatment (day 0). The low but measurable persistent viremia in these animals allowed evaluation of potential changes in plasma VL during and after treatment. Plasma VL was measured at the study entry during the chronic phase of infection (when animals were enrolled in the protocol); 0–3 days before hetIL-15 treatment (day 0); at 1 week of treatment; and at the end of the treatment (day 15, necropsy) (Table 2). Comparing day 0 to day 15 showed a decrease in VL (>15-fold) upon hetIL-15 treatment in 6 animals. The rest of the animals had smaller changes in PVL (below 10 fold). Two animals had increases (5 fold and 1.3 fold for T421 and R591, respectively) and five animals had decreases (3–7 fold). Our results do not support a strong latent virus activating effect by hetIL-15 *in vivo*, rather, the results support the conclusion that the net effect of hetIL-15 treatment was decreased virus RNA in the plasma and in LN.

**Table 2:** Plasma viral load of 13 SHIV^+^ macaques upon hetIL-15 treatment.

Macaque:	T413	T416	T419	T421	T422	T427	5726	5751	5757	5787	P917	R591	T605
**SHIV:**	1157	1157	1157	1157	1157	1157	327C	327C	327C	327C	SF162	SF162	CH505
**Plasma VL:**													
**study entry**	950	150	150	400	1700	25	200	400	200	250	380	1100	2300
**day 0, pre**	4300	40	350	2300	3600	12	30	880	480	50	160	108	1950
**hetIL-15, week 1**	1800	25	30	3500	1000	5	20	20	500	60	31	170	450
**week 2 (necropsy)**	1500	15	20	11000	230	3	10	5	5	7	3	140	120
**VL fold drop, day 0—week 2**	3x	3x	18x	0.2x	16x	4x	3x	176x	96x	7x	53x	0.8x	16x

## Discussion

We report that hetIL-15 treatment changes the lymphocyte populations within LN and significantly increases the number of potentially cytotoxic GrzB^+^CD8^+^ T cells within B cell follicles, a finding associated with reduced levels of viral RNA within LN. Follicular helper CD4^+^ T cells are critical targets for HIV infection, represent a major compartment for residual virus under conditions of immunologic or pharmacologic control of viral replication, and constitute a long-term reservoir of chronically infected cells in HIV^+^ patients and in SIV^+^ macaques, including elite controllers and persons receiving ART. In elite controller rhesus macaques infected with SIVmac239, cellular immune responses targeting the virus were very effective in eliminating most infected CD4^+^ T cells within LN except for the T_FH_ population within the follicles [36]. These results together with reports of low CTL density in areas of HIV replication within the LN [52–55] are consistent with the interpretation that B cell follicles represent an immune privileged site where infected T_FH_ cells are shielded from cytotoxic T cells capable of eliminating infected cells elsewhere. Recent studies have shown the presence of scattered CD8^+^ T cells within the B cell follicles of LN in HIV-infected patients [55, 66] and SIV infected macaques [52]. Although these studies did not address the granzyme content and cytotoxic capacity of such cells *in vivo*, these cells maintained their capacity for *in vitro* killing of infected CD4^+^ cells. The finding that hetIL-15 increased the frequency of effector CD8^+^ T cells (including Gag CM9-specific Ki67^+^GrzB^+^ cells) with cytotoxic phenotype within the LN and the follicles suggests a potential role for the use of hetIL-15 for therapeutic interventions against HIV aiming to reduce the virus reservoir. The strong induction of cytolytic molecules (granzyme, perforin) and the demonstration that CD8^+^ cells produce multiple cytokines and degranulate upon specific TCR stimulation ex vivo demonstrate that hetIL-15 enhances the cytolytic ability of CD8^+^ T cells. The immunohistochemistry results show that follicular CD8^+^ T cells also have high granzyme. By boosting the frequency of follicular CD8^+^ T cells and increasing their cytotoxicity, hetIL-15 potentially increases the possibility of follicular CD8^+^ T cells to find and eliminate infected cells in such environment. The decrease in cell-associated viral RNA in both LN and blood together with the decrease in plasma viral load suggests the elimination of virus producing cells. An alternative hypothesis that cannot be excluded at present, is that virus-producing CD4 cells may leave the LN. Infected CD4 cells leaving the LN sanctuary may become more vulnerable to elimination. The decrease in plasma VL and cell associated viral RNA suggests that, even if infected CD4 cells change location, hetIL-15 treatment results in their elimination. Lack of significant changes in the composition of CD4 T cell subsets in the LN (S3 Fig) does not support the preferential exit of infected CD4^+^ cells.

It has been reported that IL-15 can act as a HIV latency reversing agent *in vitro*, especially in synergy with other compounds [67, 68]. Our results do not provide evidence for such an activity for hetIL-15 *in vivo*, although this could be the result of low activation that is not reflected in the blood, or that is rapidly masked by the elimination of infected cells. In any case, there may be advantages to combining hetIL-15 treatment with latency reversing agents for targeting long-term viral reservoirs, including latently infected cells. hetIL-15 treatment resulted in reduction of plasma viremia and cell-associated viral RNA in LN samples without significantly affecting the levels of viral DNA. Only a fraction of the SIV DNA signal detected in the quantitative PCR assay is expected to represent replication competent proviruses [65]. This may affect the number of infected cells susceptible to immune mediated clearance dependent on expression of viral antigens.

The most dramatic decrease of LN viral RNA upon hetIL-15 treatment was observed in axillary LN, which were the draining LN for the site of SC hetIL-15 administrations. This suggests that more prolonged treatment or a different schedule or route of administration may further increase the beneficial effects of hetIL-15. The low toxicity of the optimized regimen described here indicates that additional regimens can be tested and opens the possibility for combinations with other interventions. Further studies, including more sustained treatment, will be required to monitor the long-term effects of hetIL-15 on virus levels in different compartments, in order to maximize effects on virus in reservoir sites. In this context, use of macaque hetIL-15 described in this report allows long-term treatment and facilitates future macaque studies. The observed reduction in viral RNA in LN indicates that hetIL-15 mediated effects provide new opportunities for exposure of HIV/SIV infected cells to immune clearance.

The ability of hetIL-15 to increase the number of cytotoxic CD8^+^ T cells within secondary lymphoid tissues makes the cytokine an interesting potential component in combination therapeutic interventions aiming to reduce or eradicate persistent viral reservoirs in secondary lymphoid tissues. Interventions that facilitate targeting of infected cells in the follicles may become an important component of combinatorial treatment to reduce or eliminate viral reservoirs and sanctuaries. Long-term studies in ART-treated macaques and measurements of virus reservoirs in additional nonhuman primate models of AIDS virus infection will be required to further evaluate the effects and therapeutic potential of hetIL-15 in treating chronic viral infection. The present results establish that hetIL-15 can be safely delivered and is equally effective in increasing cytotoxic cells in B cell follicles in uninfected and SHIV infected, as well as in chronically SIV-infected cART-treated macaques. hetIL-15 is presently being evaluated in clinical trials for cancer immunotherapy due to its ability to increase effector lymphocytes in tumors, and to delay tumor growth in animal models. Its ability to increase infiltration of B cell follicles by CD8^+^ may also be beneficial for other viral infections and malignancies affecting the LN.

## Materials and methods

### Ethics statement

All animals were cared for and procedures performed under a protocol approved by the Institutional Animal Care and Use Committee of BIOQUAL, Inc. (animal welfare assurance no. A3086-01; protocol numbers 14-A478-11 and 17–024) and USDA Certificate number 51-R0036. Furthermore, the macaques in this study were managed accordingly to the animal husbandry program. which aims at providing consistent and excellent care to nonhuman primates at the vivarium. This program operates based on the laws, regulations, and guidelines promulgated by the United States Department of Agriculture (e.g., the Animal Welfare Act and its regulations, and the Animal Care Policy Manual), Institute for Laboratory Animal Research (e.g., Guide for the Care and Use of Laboratory Animals, 8th edition), Public Health Service, National Research Council, Centers for Disease Control, and the Association for Assessment and Accreditation of Laboratory Animal Care (AAALAC) International. The nutritional plan utilized by the BIOQUAL, Inc. Facility consisted of twice daily feeding of Labdiet 5045 High Protein Primate Diet and food intake was closely monitored by Animal Research Technicians. This diet was also supplemented with a variety of fruits, vegetables, and other edible objects as part of the environmental enrichment program established by the Veterinary staff and enrichment Technician. Pairing of animals as part of the environmental enrichment program was managed by the enrichment technician. All primary enclosures and animal rooms were cleaned daily with water and sanitized at least once every two weeks. Macaques (N = 24) used in this study were 9 males and 15 females. Their average weight was 6 kg (range: 3.4–10.2 kg) and their average age was 8 years (range: 4–15 years). Vaccinations were performed under anesthesia (Ketamine administered at 10 mg/kg) and all efforts were made to minimize suffering. No adverse effects were found. All animals were euthanized as part of this study.

### Experimental design

The objectives of the study were to determine the safety and in vivo bioactivity of a step-dose hetIL-15 treatment delivered subcutaneously in rhesus macaques, focusing on the effects on cytotoxic lymphocytes within secondary lymphoid tissue, especially in B cell follicles, an immune privileged area where chronically HIV-infected CD4^+^ T cells are located. We assessed the impact of treatment on immunologic parameters in uninfected animals and in SHIV-infected macaques that had spontaneously controlled infection and were maintaining low level plasma viremia which also allowed assessment of virologic parameters. Lymph node, blood, and mucosal samples were analyzed before and after hetIL-15 treatment by flow cytometry, multiparameter flow cytometry, quantitative multiplexed confocal imaging (histo-cytometry) and quantitative PCR/quantitative RT PCR.

### Macaques and hetIL-15 treatment

The study was carried out in accordance with the Guide for the Care and Use of Laboratory Animals of the National Institutes of Health. Indian rhesus macaques (*Macaca mulatta*) were housed and handled in accordance and approval by the Institutional Animal Care and Use Committee of BIOQUAL, Inc. The cohort of macaques described in Table 1 consists of 9 uninfected animals and 15 (SHIV^+^) animals chronically infected with clade B SHIV (SHIV-SF162 [69]) or clade C SHIV [(SHIV CH505 [70], SHIV-1157ipd3N4 [71], SHIV-327C [72]]. The SHIV^+^ macaques were asymptomatic with low levels of persistent chronic viremia. Twenty-two animals received six SC injections during a two-week treatment cycle (day 1, 3, 5, 8, 10, and 12) with increasing doses of hetIL-15 (2, 4, 8, 16, 32 and 64 μg/kg, “step dose”). Two animals received two-week treatment cycle of hetIL-15 using either a dose-escalation of 5–120 μg/kg (P934) or repeated administration of 50 μg/kg (P995). We took advantage of the role of hetIL-15 as homeostatic cytokine [57, 58] to develop the step-dose treatment regimen, which provides increasing doses of the cytokine as the lymphocytes divide and increase in numbers. This allowed optimal lymphocyte expansion with lower drug exposure. hetIL-15 treatment was well tolerated and the macaques did not have any clinically apparent adverse effects or laboratory abnormalities described with other IL-15 treatment regimens [28]. Animals treated with the step dose protocol did not develop edema, fever or low blood pressure. Several had increases in the size of axillary and inguinal LN.

Eight of the 24 macaques were treated with macaque hetIL-15; the remainder received the human molecule (Table 1). Human and macaque hetIL-15 were produced in HEK293 cells (Invitrogen) and purified as described [19, 24], except that crude macaque hetIL-15 was subjected to two additional steps, first concentration using tandem tangential flow filtration (TangenX) and then anion exchange chromatography using Capto Q resin (GE Healthcare Science). Human and macaque purified hetIL-15 cytokines were equipotent in macaque cells *in vitro* (S1 Fig). All hetIL-15 concentrations are given as single-chain IL-15 polypeptide mass equivalents within the heterodimer. Three days before treatment initiation and 3 days after the last hetIL-15 injection, peripheral LN, peripheral blood, vagina, and rectum samples were collected, lymphocytes were isolated and analyzed as previously described [73].

Two macaques (R905, R913) infected rectally with repeated low dose of SIVmac251 and treated with combination antiretroviral therapy (cART) were used to compare the effects of hetIL-15 with the uninfected and SHIV infected macaques. At weeks 10–12 postinfection, the animals were placed on cART given in a single formulation s.c. daily at 1 ml/kg comprising Tenofovir [PMPA] (20 mg/kg), Emcitrabine [FTC] (50 mg/kg) and Dolutegravir [DTG] (2.5 mg/ml) (Gilead and ViiV). The animals maintained plasma VL below 50 copies/ml during 35 weeks in cART and were treated twice with the step-dose hetIL-15 two-week regimen, with a 4-week rest period.

### Immune phenotyping and flow cytometry

The following fluorophore conjugated monoclonal antibodies were used: CD3 (clone SP34-2), CD4 (clone L200), CD95 (clone DX2), Ki67 (clone B56), CD16 (clone 3G8), γδ TCR (clone B1), IFN-© (clone B27), TNFα (clone Mab11) (BD Biosciences), Perforin (clone Pf-344) (Mabtech), CD107a (clone eBioH4A3), CD28 (clone CD28.2); CD8 (clone 3B5) and Granzyme B (clone GB12; Life Technologies). Briefly, lymphocyte single cell suspensions were washed with PBS supplemented with 0.2% heat-inactivated human serum (Sigma), and incubated with different cocktails of fluorophore-labelled monoclonal antibodies during 20 minutes at room temperature [73], fixed and permeabilized using the FoxP3 permeabilization reagent (eBioscience). After 30 minutes incubation at 4°C, the cells were washed with FoxP3 washing buffer and intracellularly stained with Ki67 and GrzB for 20 minutes. The cells were washed and resuspended in PBS for flow cytometry analysis. For tetramer staining using samples from MamuA*01^+^ macaques, the CM9 tetramer was added to the samples 5 minutes prior to the addition of the antibody cocktail for surface staining [73]. The samples were acquired on a Fortessa or LSRII flow cytometer (BD Biosciences, San Jose, CA) and the data were analyzed using the FlowJo software platform (Tree Star, Inc., Ashland, OR).

### Cytotoxic responses

Two different assays were used to monitor cytotoxic responses by cryopreserved CD8^+^ lymphocytes from MamuA*01^+^ rhesus macaques: degranulation and direct target cell killing. For the degranulation assay, PBMC and LNMC were thawed and incubated in RPMI supplemented with 10% FBS, antibiotics and 30 units/ml of recombinant DNase I. After 24 hours, the cells were washed, seeded in 96-well plates (3x10^5^ cells/well) and stimulated with the MamuA*01-restricted SIV Gag_181-189_ CM9 peptide at a final concentration of 1μg/ml in the presence of Monensin. As negative control, cells were also stimulated with a pool of peptides (15-mers overlapping by 11 aa) covering HIV-1 Vif. At different time points, samples were washed, stained with the CM9 tetramer followed by a surface antibody cocktail containing CD3, CD4, CD8 and CD107a to monitor degranulation and release of cytotoxic enzymes. After permeabilization, the cells were intracellularly stained with antibodies for IFN-©, TNFα, granzyme B and perforin.

Direct target cell killing: autogous bone marrow cells were used as targets. Briefly, the cells were labeled with CFSE at a concentration of 1 μM for 30 minutes at 37°C. After the incubation, the cells were washed with PBS to remove the excess of dye and cultured overnight at 37°C. Next day, the cells were loaded with the Gag_181-189_ CM9 peptide at a concentration of 5 μg/ml. After 30 minutes, the cells were washed and mixed with autologous LNMC at a ratio of 1:1. After 2 hours at 37°C, the fluorescent 660-DEVD-FMK reagent, an inhibitor of caspase 3 and caspase 7 that difuses into the cells and irreversibly binds to the active forms of the caspases (FLICA assay, ImmunoChemistry Technologies) was added to the samples. After 1 hour, the samples were washed with the FLICA kit buffer and analyzed for apoptosis by flow cytometry.

### Multiparameter confocal imaging

Confocal imaging was performed with formalin fixed paraffin embedded (FFPE) lymph node sections ~10 μm in thickness. Tissue sections were deparaffinized by bathing in xylene and serial ethanol dilutions. Antigen retrieval was performed at 110°C for 15 minutes using Borg RTU (Biocare Medical). Tissue sections were then blocked, permeabilized for 1 hour at room temperature and stained with the following primary and conjugated antibodies: anti-CD20 Pacific Blue (in house conjugated) (clone L26), anti-CD3 Alexa Fluor 680 or 594 (clone F7.2.38), anti-CD4 Alexa Fluor 488 (goat polyclonal IgG, FAB8165G, R&D systems), anti-Granzyme B Alexa Fluor 647 or 680 (clone GrB-7), anti-Ki67 Brilliant Violet 421 or 510 (clone B56) and the nuclear marker JOJO-1 (Life Technologies). Stainings were carried out consecutively with the primary antibodies being added first and incubated overnight at 4° C, followed by staining with the appropriate secondary antibody Alexa Fluor 647 for PD-1 (goat polyclonal, BAF1086, R&D). Conjugated antibody stainings were performed for 2 hours at room temperature, after which sections were stained with Jo-Pro-1 for nucleus identification. Images were acquired at a 512 x 512 pixel density using a 40x objective (NA 1.3) either on a Leica TCS SP8 confocal platform running LAS-X or a NIKON C2si confocal microscope running NIS-elements AR. Depending on the platform, fluorophore spectral spillover was corrected either by acquiring single stained tissue controls and calculating a spillover matrix that was then used to correct for cross-talk (Leica) or through live spectral un-mixing (NIKON).

### Histo-cytometry and imaging analysis

Post-acquisition analysis was performed using the Imaris software (Bitplane, version 8.3.1). Histo-cytometry was performed as previously described [55, 63]. Briefly, 3-TFH dataset dimensional imaging datasets were segmented based on their nuclear staining signal and average voxel intensities for all channels were extrapolated in Imaris after iso-surface generation. Data were then exported to Microsoft Excel, concatenated into a single comma separated values (cmv) format and imported into FlowJo version 10 for further analysis. To account for variations in the size of tissues screened, data were normalized to cells/mm^2^ follicle area. Areas were calculated in Imaris post-acquisition using the iso-surface generation modality.

### Virus RNA and DNA measurements

Plasma SIV *gag* RNA and cell associated SIV *gag* DNA and RNA in LN and PBMC were measured using quantitative PCR and RT PCR methods, essentially as described using high sensitivity assay formats [70, 74].

### Statistical analysis

Statistical analyses were performed using Prism version 7 (Graph Pad software) or SAS. Comparisons were done using nonparametric t tests, as appropriate and ANOVA. For the analysis of data in Fig 4E, a Box-Cox power transformation was applied to the data before analysis to meet the normality assumption of the model. Two-way ANOVA was used for the SHIV+ versus uninfected and pre- versus post-hetIL-15 effects, with random effects to account for the clustered values by animal.

## Supporting information

S1 Fig Human and macaque hetIL-15 are equipotent in primary macaque cells *in vitro*. Macaque PBMC were cultured in the presence of different concentrations (from 0.15 to 160 ng/ml) of either macaque or human hetIL-15. After six days, cytokine bioactivity was assessed by cell phenotyping and detection of Ki67 in the different samples. Human and macaque purified hetIL-15 cytokines are equipotent in primary macaque cells *in vitro*, showing a hierarchical response with NK>CD8^+^ T cells >CD4^+^ T cells.
(TIF)

S2 Fig Flow cytometry gating strategy. Dot plots showing the gating strategy for the lymphocyte analysis after acquisition. Singlets were identified based in the FSC-A and FSC-H properties followed by a live/dead gate using a fixable viability dye. A gate based in forward and side scatter was used to determine the lymphocyte population among the live cells. T cells were identified within the lymphocyte gate, and further subdivided in the CD4^+^ and CD8^+^ T cells subsets. Memory cell subsets were identified using the CD95 and CD28 antibody combination: naïve (CD95^-^CD28^+^), central memory (T_CM_, CD95^+^CD28^+^) and effector memory (T_EM_, CD95^+^CD28^-^). NK cells were identified in the CD3^-^ population using the markers CD16 and GrzB. In each individual T cell subset, the cytotoxic and proliferating populations were identified by the use of GrzB or Ki67, respectively.
(TIF)

S3 Fig CD4^+^ lymphocyte subsets in LN measured by flow cytometry of LNMC of the sixteen animals in Fig 1. No statistical differences were detected before and after hetIL-15.
(TIF)

S4 Fig Multiparametric confocal imaging and histo-cytometry analysis. (A) Antibody panel used for tissue immunophenotyping. Confocal imaging was performed using ~5 μm sections of formalin fixed paraffin embedded (FFPE) lymph nodes. (B) Histo-cytometry was performed as previously described [55, 63]. Histo-cytometry derived 2D plots show the gating strategy for identification of relevant populations. Sphericity and volume coordinates were used for identification of cell events and the combination of CD20^hi/dim^Ki67^hi^ for the identification of follicular and GC areas. Individual follicular areas, encircled by dotted lines, are shown on the X-Y plot. Examples of the frequency of CD8^+^ cells (defined as CD3^hi^CD4^lo^) and CD8^+^GrzB^+^ cells in individual follicles are shown (lower panel).
(TIF)

S5 Fig Comparison of CD8^+^GrzB^+^ cells in B cell follicles of two SIV-infected cART-treated macaques before and after hetIL-15. Animals were treated daily with cART and were aviremic (<50 copies of viral RNA/ml plasma). They received two cycles of hetIL-15 treatment of two week duration with a four week rest between the cycles. The number of CD8^+^ GrzB^+^ cells in B cell follicles is normalized to the total LN tissue area.
(TIF)
