# Correction: Treatment with native heterodimeric IL-15 increases cytotoxic lymphocytes and reduces SHIV RNA in lymph nodes

**DOI:** 10.1371/journal.ppat.1007345

**Published:** 2018-10-11

**Authors:** Dionysios C. Watson, Eirini Moysi, Antonio Valentin, Cristina Bergamaschi, Santhi Devasundaram, Sotirios P. Fortis, Jenifer Bear, Elena Chertova, Julian Bess, Ray Sowder, David J. Venzon, Claire Deleage, Jacob D. Estes, Jeffrey D. Lifson, Constantinos Petrovas, Barbara K. Felber, George N. Pavlakis

An affiliation is missing for the first author. In addition to the current affiliation information, Dionysios C. Watson is affiliated with: Department of Medicine, University of Patras, Greece.
