# Supplementary figures and images for: Treatment with native heterodimeric IL-15 increases cytotoxic lymphocytes and reduces SHIV RNA in lymph nodes
Source: PLoS Pathog. 2018 Feb 23;14(2):e1006902. doi: 10.1371/journal.ppat.1006902 (PMC5825155; doi:10.1371/journal.ppat.1006902)

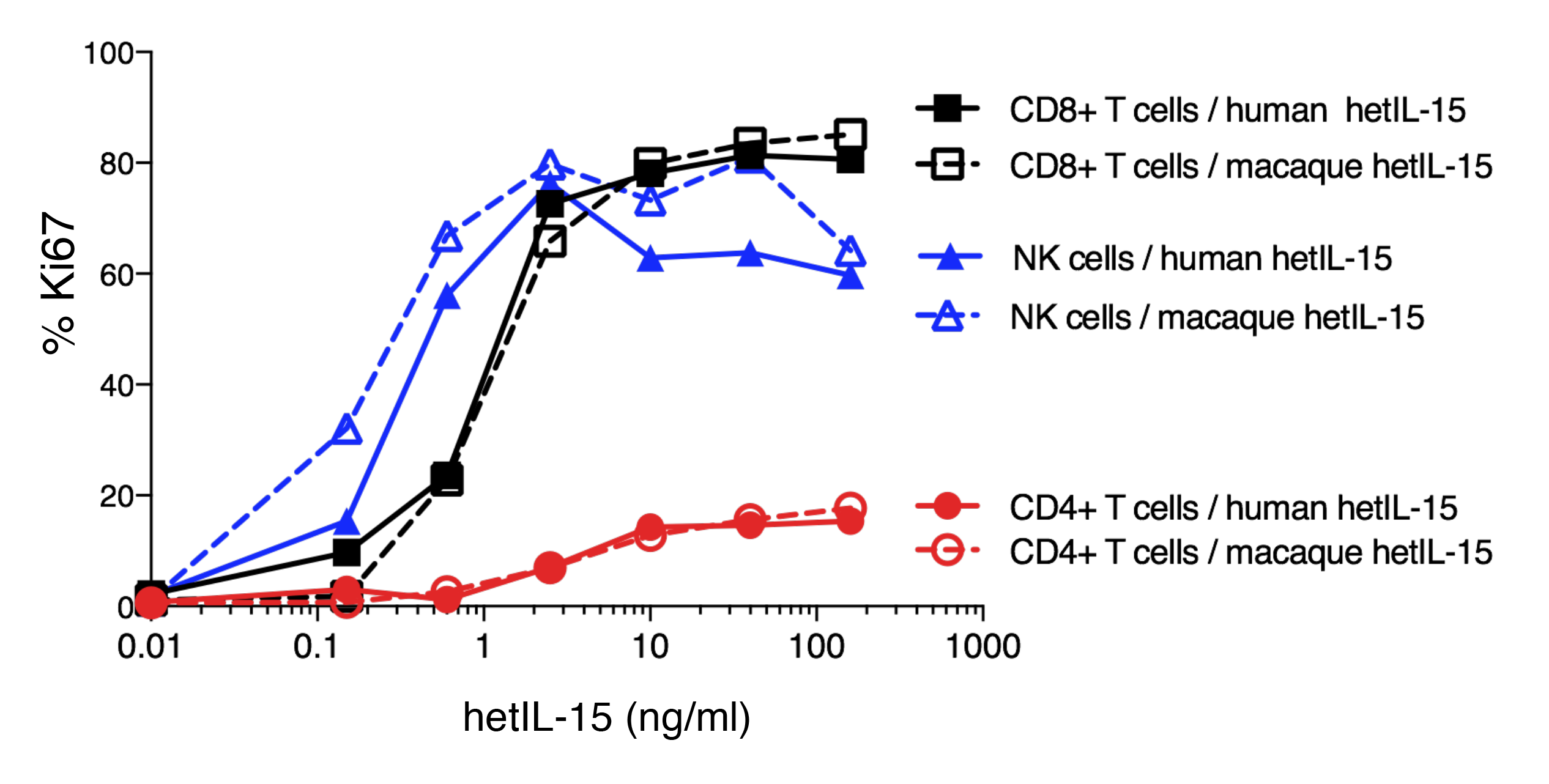

Supplement: S1 Fig — Macaque PBMC were cultured in the presence of different concentrations (from 0.15 to 160 ng/ml) of either macaque or human hetIL-15. After six days, cytokine bioactivity was assessed by cell phenotyping and detection of Ki67 in the different samples. Human and macaque purified hetIL-15 cytokines are equipotent in primary macaque cells in vitro, showing a hierarchical response with NK>CD8+ T cells >CD4+ T cells. (TIF) [file ppat.1006902.s001.tif]

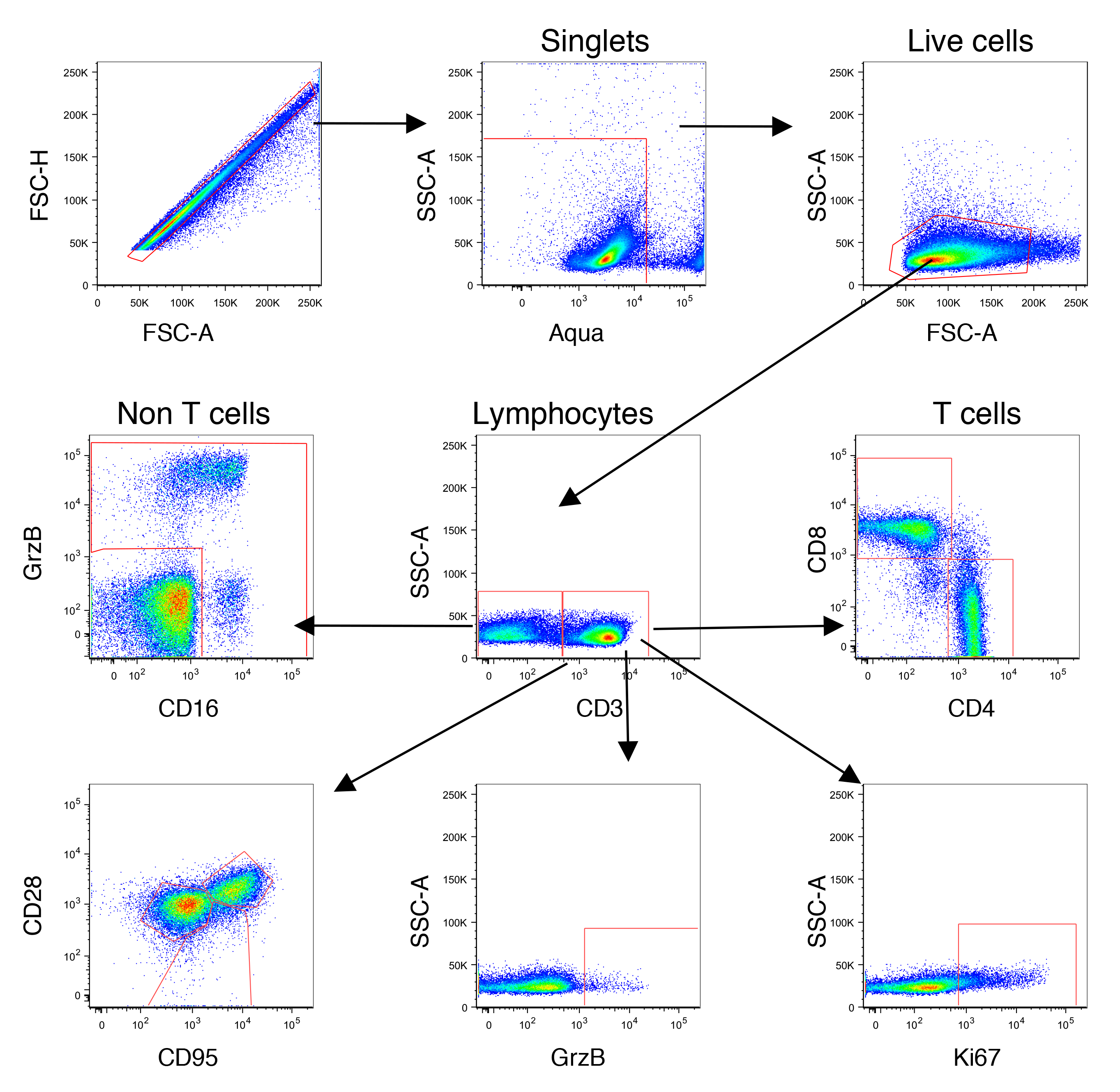

Supplement: S2 Fig — Dot plots showing the gating strategy for the lymphocyte analysis after acquisition. Singlets were identified based in the FSC-A and FSC-H properties followed by a live/dead gate using a fixable viability dye. A gate based in forward and side scatter was used to determine the lymphocyte population among the live cells. T cells were identified within the lymphocyte gate, and further subdivided in the CD4+ and CD8+ T cells subsets. Memory cell subsets were identified using the CD95 and CD28 antibody combination: naïve (CD95-CD28+), central memory (TCM, CD95+CD28+) and effector memory (TEM, CD95+CD28-). NK cells were identified in the CD3- population using the markers CD16 and GrzB. In each individual T cell subset, the cytotoxic and proliferating populations were identified by the use of GrzB or Ki67, respectively. (TIF) [file ppat.1006902.s002.tif]

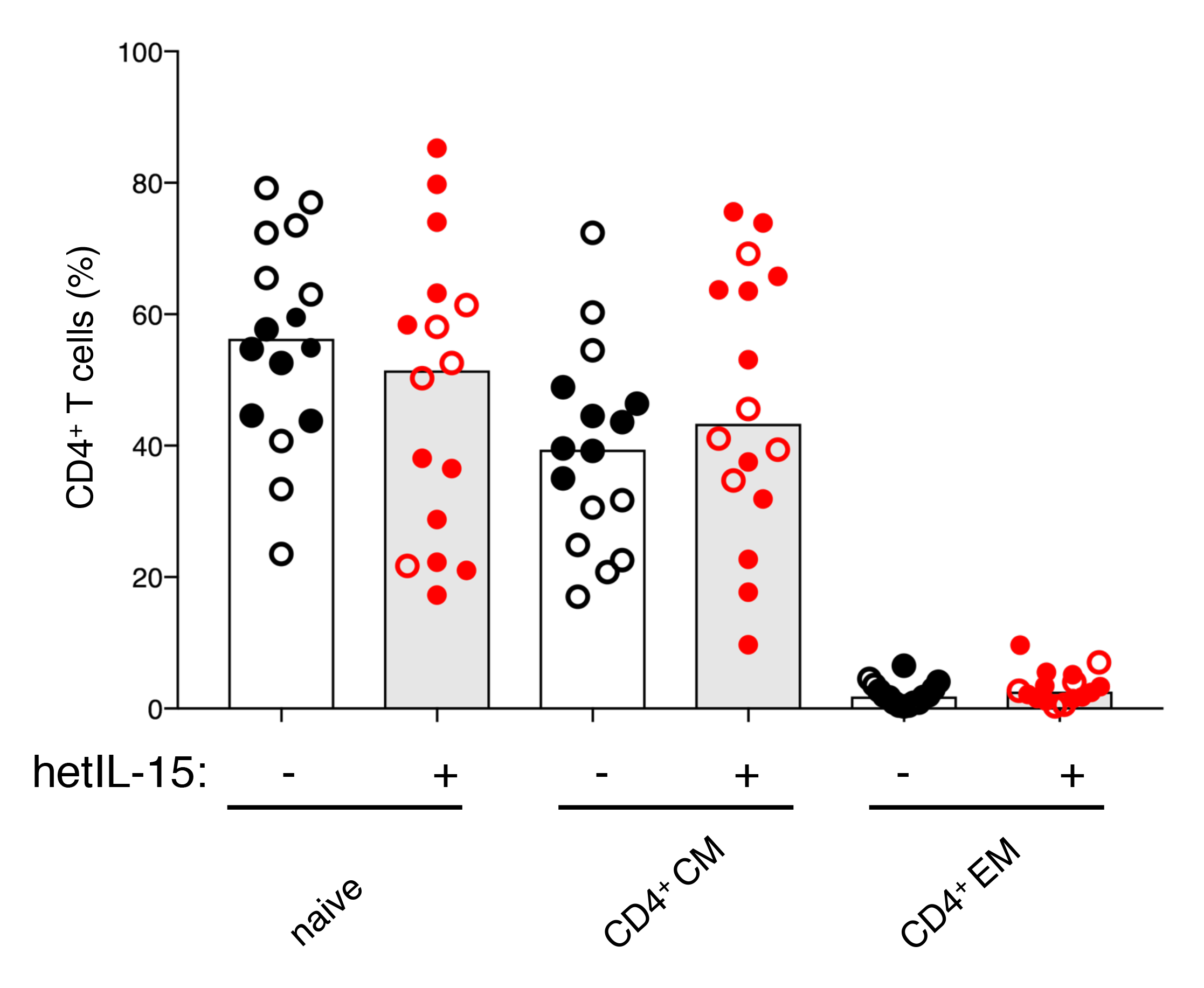

Supplement: S3 Fig — No statistical differences were detected before and after hetIL-15. (TIF) [file ppat.1006902.s003.tif]

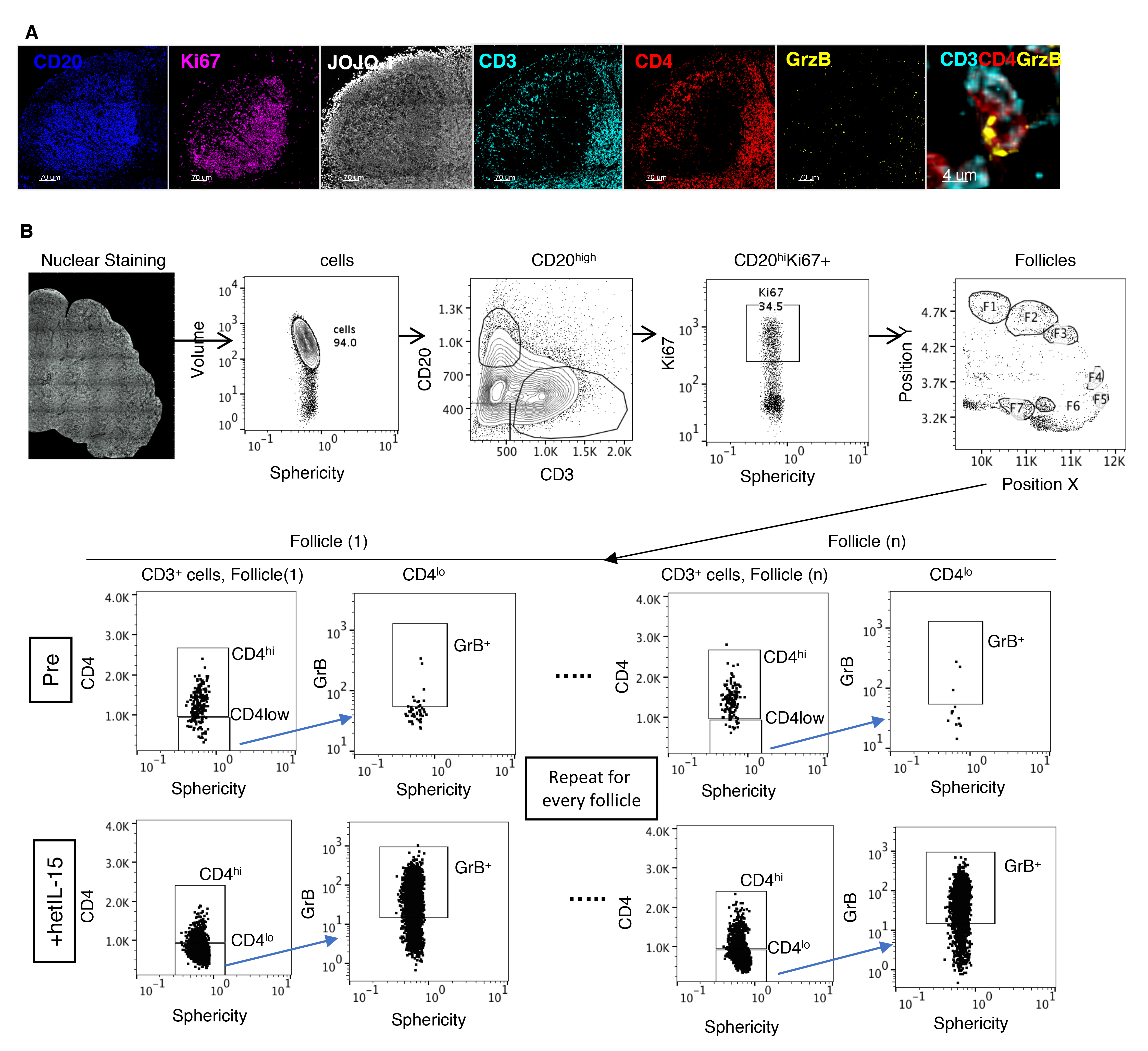

Supplement: S4 Fig — (A) Antibody panel used for tissue immunophenotyping. Confocal imaging was performed using ~5 μm sections of formalin fixed paraffin embedded (FFPE) lymph nodes. (B) Histo-cytometry was performed as previously described [55, 63]. Histo-cytometry derived 2D plots show the gating strategy for identification of relevant populations. Sphericity and volume coordinates were used for identification of cell events and the combination of CD20hi/dimKi67hi for the identification of follicular and GC areas. Individual follicular areas, encircled by dotted lines, are shown on the X-Y plot. Examples of the frequency of CD8+ cells (defined as CD3hiCD4lo) and CD8+GrzB+ cells in individual follicles are shown (lower panel). (TIF) [file ppat.1006902.s004.tif]

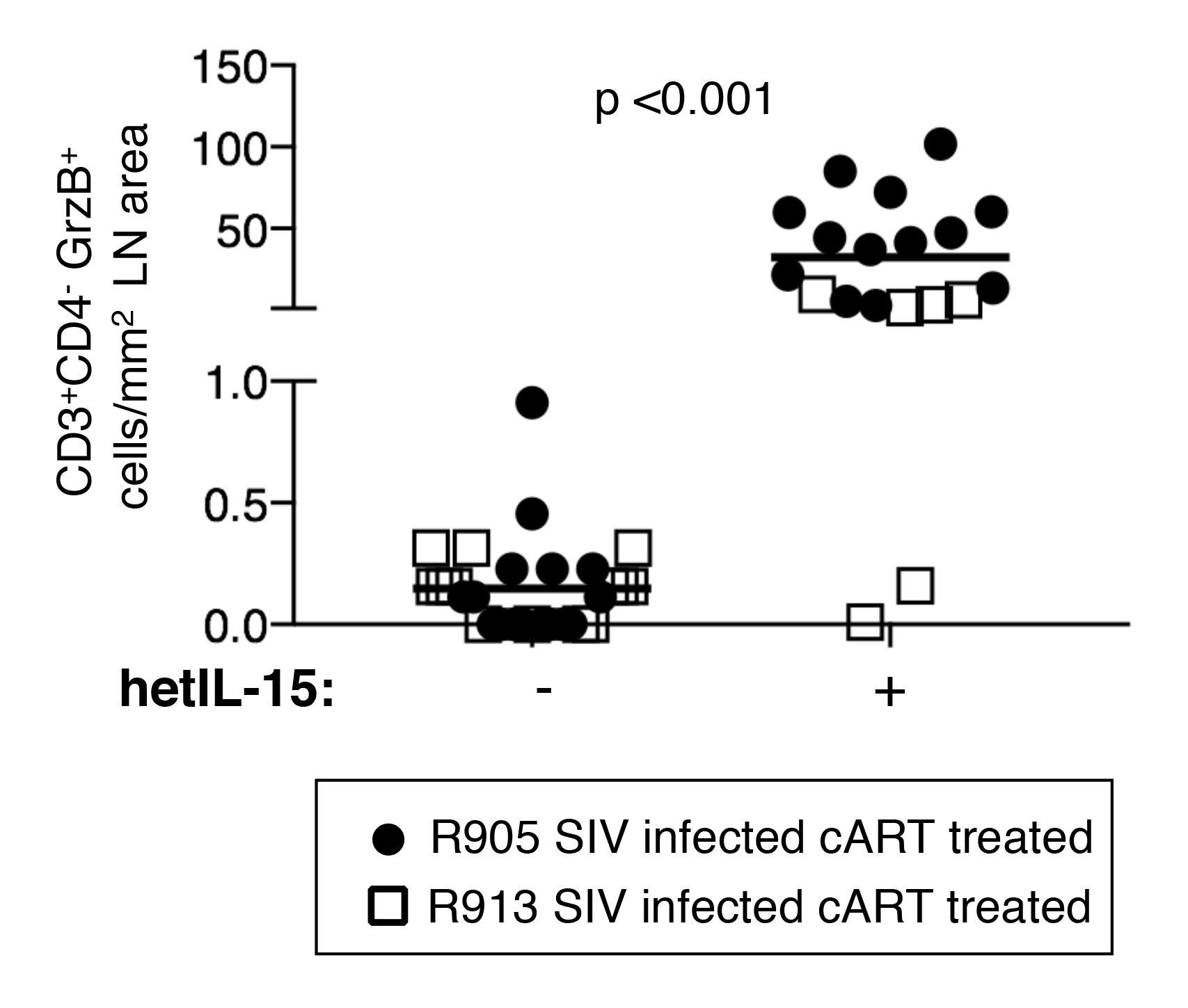

Supplement: S5 Fig — Animals were treated daily with cART and were aviremic (<50 copies of viral RNA/ml plasma). They received two cycles of hetIL-15 treatment of two week duration with a four week rest between the cycles. The number of CD8+ GrzB+ cells in B cell follicles is normalized to the total LN tissue area. (TIF) [file ppat.1006902.s005.tif]
